# Do Leptin Play a Role in Metabolism–Related Psychopathological Symptoms?

**DOI:** 10.3389/fpsyt.2021.710498

**Published:** 2021-09-10

**Authors:** Yelei Zhang, Xiaoyue Li, Xianhu Yao, Yating Yang, Xiaoshuai Ning, Tongtong Zhao, Lei Xia, Yulong Zhang, Kai Zhang, Huanzhong Liu

**Affiliations:** ^1^Department of Psychiatry, Chaohu Hospital, Anhui Medical University, Hefei, China; ^2^School of Mental Health and Psychological Sciences, Anhui Medical University, Hefei, China; ^3^Maanshan Fourth People's Hospital, Maanshan, China

**Keywords:** leptin, body mass index, psychopathological symptoms, schizophrenia, antipsychotics

## Abstract

**Objectives:** Leptin is a crucial regulator of energy balance and is associated with obesity. In recent years, it has also been recognized as involved in the psychopathological mechanism. Our study aimed to elucidate the relationships between serum leptin levels, body mass index (BMI), and psychopathology symptoms in patients with schizophrenia.

**Methods:** A cross-sectional assessment of 324 inpatients with schizophrenia was conducted. Schizophrenia symptoms were measured using the Positive and Negative Syndrome Scale (PANSS) and the Brief Psychiatric Rating Scale (BPRS). Serum leptin levels were assessed by the Enzyme-Linked Immunosorbent Assay (ELISA).

**Results:** Significant differences in sex, BMI, and negative symptom subscale (PANSS-N) scores were found between the groups with high and low leptin levels in the study. Leptin levels were positively correlated with BMI (B = 2.322, *t* = 9.557, *P* < 0.001) and negatively correlated with PANSS-N scores (B = −0.303, *t* = −2.784, *P* = 0.006).

**Conclusions:** Our results suggest that the increase in leptin levels is responsible for antipsychotic-induced weight gain and improved psychopathological symptoms.

## Introduction

Schizophrenia is a chronic syndrome with a variety of clinical symptoms and biological characteristics (1), and its symptoms are usually divided into three categories (general psychopathological symptoms and positive and negative symptoms) (2). The life expectancy of people with schizophrenia is about 11–20 years shorter than that of the general population, and the average life expectancy is about 80–85% of that of the general population. Studies show that most people with schizophrenia die from complications such as cardiovascular disease. This phenomenon is mainly due to the higher risk of weight gain and other adverse metabolic effects caused by antipsychotics in patients with schizophrenia (3–5). Although the pathophysiological mechanisms of metabolic disorders, including weight gain induced by antipsychotics, remain unclear, associations between metabolic changes and psychopathological symptoms have been reported (6).

With the increasing use of second-generation antipsychotics (SGA), metabolic side effects are becoming more common, such as weight gain, changes in blood lipids, and glucose intolerance (7). Among second-generation antipsychotics, weight gain is the most common metabolic side effect in patients with schizophrenia who take these medications. The obesity rate is about 26–55% of mental disorders, 4.3 times higher than that of the general population (8). A meta-analysis comparing the efficacy of antipsychotics found that clozapine and olanzapine, while more effective than other drugs, were also more likely to cause weight gain than other drugs (9). Furthermore, the weight gain induced by olanzapine was dose-dependent in the short term after treatment (10). There is some evidence that antipsychotics with a faster track of weight gain during early treatment (such as olanzapine and clozapine) are more likely to gain weight than drugs with a slower track of weight gain. This phenomenon is significantly associated with clinical efficacy (11, 12). Ziprasidone and aripiprazole were also associated with weight gain in patients with schizophrenia who were first treated with antipsychotics (13, 14). However, the weight gain effect induced by aripiprazole was significantly lower than that of olanzapine (15). Pimavanserin is adjuvant clozapine, can significantly increase the weight of patients (16, 17). In addition, first-generation antipsychotics (FGA), such as chlorpromazine and thiouracil, have also been found to cause significant weight gain (18). Therefore, there is reason to suspect that the early clinical efficacy of the drug is associated with an increased cardiovascular burden.

Weight gain caused by medication, if not treated and managed, may lead to more serious consequences, that is, metabolic syndrome (19). Although clozapine and olanzapine have unique advantages in treating refractory schizophrenia, they are most likely to cause metabolic abnormalities (20). Clozapine and olanzapine also directly increase the risk of hyperlipidemia and hypertension and are unrelated to their effects on obesity and glucose tolerance (21, 22). Clozapine, in particular, increases the above risks (23). These risks will increase rapidly in a short time after treatment, endangering the lives of patients (24).

If there is a correlation between antipsychotic-induced weight gain and treatment effectiveness, the nature of the relationship is worth exploring. There are three hypotheses for this link:

1. The weight gain of patients directly improves the symptoms during treatment.
2. Clinical efficacy will lead to weight gain.
3. Antipsychotics induce weight gain and symptom improvement in patients in (a) interdependent/interdependent or (b) independent/mutually exclusive manner.

This means:

1. Antipsychotic-induced weight gain may be necessary for treatment effectiveness.
2. Weight gain caused by antipsychotics is a side effect that can be safely targeted without affecting the final therapeutic effect (e.g., lifestyle or drug intervention).

Leptin is an anorexic hormone produced by adipocytes and whose levels increase with weight gain (25). The energy-related role of leptin has been widely studied. As a satiety factor, leptin plays a vital role in maintaining energy balance by interacting with neural pathways in the brain, especially those involving the hypothalamus (26). Moreover, there was evidence that leptin promotes cognitive and behavioral functions in both rodents and humans (27). Recently, attention has been focused on the effect of leptin on psychopathological symptoms in patients with schizophrenia.

Antipsychotics can increase body weight, with a concurrent increase in leptin levels. Several studies have reported a positive relationship between weight gain and clinical status improvement after treatment. In addition, elevated serum leptin levels have been positively correlated with overall psychopathological improvement and are considered a predictor of clinical improvement (28). Although there are suggestions that leptin may be a biomarker for the prognosis of schizophrenia, it remains unclear whether this hormone is associated with particular psychopathological parameters. Few studies have explored leptin changes in patients with psychopathologies, especially in Chinese patients with schizophrenia. Therefore, our research aims to explore (1) serum leptin levels in patients with schizophrenia and (2) whether there was a relationship between serum leptin levels, BMI, and psychopathological parameters.

## Materials and Methods

### Participants

We collected data on inpatients with schizophrenia from three hospitals in Anhui Province (the Chaohu Hospital of Anhui Medical University, Hefei Fourth People's Hospital, and Ma'an shan Fourth People's Hospital). The obesity rate in schizophrenia is estimated at 20% and in the general population at 10% (29), with a prevalence difference of about 10% between the two groups. Considering the following relevant parameters, α = 0.05, 1–β = 0.82, *R* = 0.5, 323 patients should be included through PASS11 calculation.

The inclusion criteria were as follows: (1) patients aged 18–75 years; (2) patients diagnosed with schizophrenia using the International Classification of Diseases, 10th Revision (ICD-10); (3) those with a disease duration of more than 5 years and was hospitalized for more than 1 month and (4) those with the ability to provide written informed consent and participate in psychopathology assessments. The exclusion criteria were as follows: (1) patients diagnosed with other mental disorders using the ICD-10; (2) those with severe physical illnesses, including severe neuroendocrine or metabolic disease; (3) those who abuse alcohol or other substances; and (4) pregnant or lactating women.

The study initially involved 443 patients, and 324 were eventually included, with an effective rate of 73.14%. Of the 119 excluded patients, 85 refused to participate in further interviews, nine refused to provide blood samples, nine were discharged from the hospital, and 16 had incomplete blood test data. In general, the study included three groups of people: (1) medically stable patients who chose to be hospitalized for family reasons; (2) patients who relapse due to irregular drug use; and (3) long-term hospitalized patients with unstable conditions. All aspects of the study protocol were reviewed and approved by the ethics. All of the participants provided written informed consent to participate in this study.

### Demographic Data

The general information was collected by query and recorded on the questionnaires by professional psychiatrists trained for the task according to a uniform set of criteria. Height (m) and weight (kg) were measured. Researchers reviewed the histories of schizophrenia inpatients. We divided the subjects into three groups according to their current medication status, including the typical antipsychotics group (such as sulpiride, haloperidol), the atypical antipsychotics group (such as olanzapine, clozapine, risperidone, quetiapine, ziprasidone, aripiprazole, amisulpride), and the combination group (use both types of antipsychotics). Using the defined daily dose (DDD) recommended by the WHO as an indicator, each drug's equivalent dose of chlorpromazine was calculated for further analysis (30).

### Clinical Assessment

To verify the diagnosis of schizophrenia, two independent psychiatrists conducted the Structured Clinical Interview for the ICD-10 with each patient. The psychopathological symptoms were evaluated using the Positive and Negative Syndrome Scale (PANSS) and the Brief Psychiatric Rating Scale (BPRS). When interrater inconsistency was observed, the raters underwent consistency training and received clarifications regarding the rating scale. The intraclass correlation coefficient (ICC) of the scores given by the four scale evaluators was 0.89. The PANSS consists of three subscales: the positive symptoms subscale (PANSS-P, items P1–P7), the negative symptoms subscale (PANSS-N, items N1–N7), and the general psychopathology symptoms subscale (PANSS-G, items G1–G16) (31).

### Measurement of Serum Leptin

All three hospitals stopped serving dinner after 6:30 p.m., but as a precaution, subjects were still asked to fast for more than 8 h before blood collection. Then, venous blood was collected between 6:00 and 8:00 a.m., and each subject's samples were stored in two 5 ml vacutainer tubes containing potassium EDTA. The plasma was labeled with anonymous codes after centrifugation (15 min at 1,000×g) and stored at −80°C. After all subjects were enrolled, the leptin levels in serum specimens were measured using an ELISA kit purchased from Cusabio Biotechnology Company (Code: CSB-E04649h). In brief, serum leptin was measured according to the instructions. The detection range was 0.156–10 ng/ml. An intra-lot coefficient of variation <8% and an inter-lot coefficient <10% were calculated.

### Statistical Analysis

All statistical analyses were calculated by SPSS 24.0 (SPSS Inc., Chicago, IL, USA), including independent sample *t*-test (normally distributed continuous variables) and Mann-Whitney *U*-test (non-normally distributed continuous variables). The chi-squared test compared the categorical variables, including sex, smoking, drinking, obesity, and current antipsychotic drug treatment. Relationships between BMI, serum leptin levels, and psychopathology symptoms were examined by Spearman's correlation analysis. Then, taking the serum leptin levels (ng/ml) as the dependent variable, multiple stepwise regression was performed to examine the relationship between serum leptin and psychopathology symptoms among participants with schizophrenia. Subsequently, linear regressions were performed to examine the relationship between leptin, BMI, negative symptoms. Moreover, multiple tests were adjusted using Bonferroni correction, and *P* < 0.05 (two-tailed) indicated statistically significant differences.

## Results

### General Information

The data of 324 inpatients with schizophrenia were analyzed, comprising 191 males and 133 females. Across all participants, the leptin data had a positively skewed distribution (skewness = 1.5, kurtosis = 2.1). Patients were grouped according to quartiles of the distribution of serum leptin levels. Those in the upper quartile were considered the high-level group (81 patients; 2.86 ng/mL), and the remainder were considered the low-level group (243 patients; 2.86 ng/mL). Table 1 shows the baseline characteristics of the two groups. The average age of study subjects was 45.06 ± 11.62 years, and the onset age was 26.06 ± 8.16 years. The disease duration was 19.01 ± 10.46 years. Moreover, there were significant differences in sex, BMI, and PANSS-N scores between the low-level and high-level groups (*P* < 0.05). The high leptin level group had a significantly higher BMI than the low leptin level group (26.67 ± 3.68 vs. 23.28 ± 3.54, *P* < 0.001). In contrast, Negative subscale scores showed lower levels in the high leptin level group (19.82 ± 7.45) than in the low leptin level group (22.10 ± 7.61). There were no differences in age of onset, disease duration, type and dose of antipsychotics between the two groups (*P* > 0.05, Table 1).

**Table 1:** Demographic characteristics of study participants.

	**Total *N* = 324**	**Low-level group *N* = 243**	**High-level group *N* = 81**	**Statistics**	***p-*value**
Age (years), Mean (SD)	45.06(11.62)	45.05(11.15)	45.09(13.02)	Z = −0.10^¶^	0.919
Gender, *n*(%)				*χ^2^* = 72.96	<0.001
Male	191(58.9%)	176(92.1%)	15(7.9%)		
Female	133(41.1%)	67(50.4%)	66(49.6%)		
Smoker, *n*(%)	94(29.0%)	88(93.6%)	6(6.4%)	*χ^2^* = 24.48	<0.001
Drinker, *n*(%)	53(16.4%)	47(88.6%)	6(11.4%)	*χ^2^* = 6.32	0.012
BMI(kg/m^2^),mean(SD)	24.12(3.86)	23.28(3.54)	26.67(3.68)	*t* = −7.38	<0.001
Obesity	129(39.8%)	74(57.4%)	55(42.6%)	*χ^2^* = 35.55	<0.001
Age of onset(years),mean(SD)	26.06(8.16)	25.69(7.96)	27.16(8.70)	Z = −1.35	0.177
Duration of illness(years), mean(SD)	19.01(10.46)	19.46(10.32)	17.67(10.82)	Z = −1.62	0.106
Current antipsychotic treatment, *n*(%)				*χ^2^* = 0.29	0.867
Typical antipsychotics	7(2.1%)	5(71.5%)	2(28.5%)		
Atypical antipsychotics	133(41.0%)	98(73.6%)	35(26.4%)		
Combination of typical and atypical antipsychotics	184(56.7%)	140(76.0%)	44(24.0%)		
Dosage of antipsychotics (mg/d)^†^,mean(SD)	455.91(258.25)	467.20(266.61)	422.04(229.58)	Z = −1.22	0.223
PANSS^‡^,mean(SD)					
Positive subscale	17.91(7.42)	18.23(7.60)	16.96(6.80)	Z = −1.14	0.255
Negative subscale	21.53(7.63)	22.10(7.61)	19.82(7.45)	*t* = 2.35	0.019
General psychopathology subscale	38.40(12.68)	39.04(12.55)	36.47(12.96)	*t* = 1.58	0.115
Total	77.81(24.18)	79.33(23.84)	73.25(24.76)	*t* = 1.97	0.050
BPRS^§^ total score	42.61(13.85)	43.40(13.77)	40.24(13.92)	Z = −1.89	0.059
Leptin levels(ng/ml), mean(SD)	1.90(1.91)	0.97(0.72)	4.72(1.55)	*t* = 21.02	<0.001

† *Chlorpromazine equivalent*.

‡ *Positive and negative syndrome scale*.

§ *BPRS, Brief Psychiatric Rating Scale*.

¶ *Mann-Whitney U*.

### The Correlations Between Leptin and Psychopathology Symptoms

**Figure 1:**
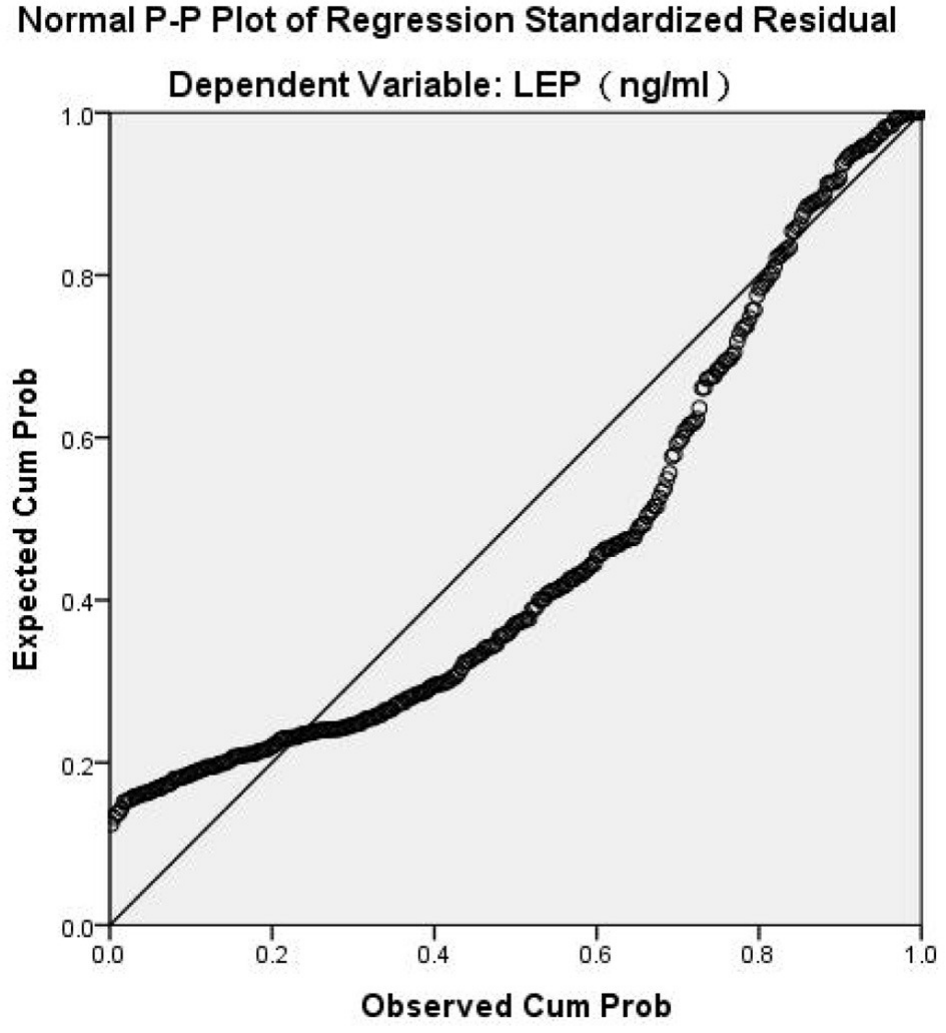
Multiple stepwise regression analysis between leptin and psychopathology symptoms.

Spearman's correlation analysis revealed that leptin was positively correlated with BMI (*r* = 0.573, *P* < 0.001) and negatively correlated with all psychopathology symptoms (*P* < 0.05). BMI was negatively associated with negative symptoms (*r* = −0.159, *P* = 0.004). BMI had no significant correlation with PANSS-P scores, PANSS-G scores, total PANSS scores, or the BPRS scores (*P* > 0.05). In general, our results indicated that leptin, BMI, and PANSS-N scores were all correlated with each other (Table 2). As shown in Table 3, Multiple stepwise regression analysis showed a negative relationship between serum leptin levels and PANSS-N (B = −0.043, *t* = −3.102, *P* = 0.002). Multiple stepwise regression analyses of leptin and psychopathic symptoms revealed that the residuals conformed to a normal distribution. It means the model was stable (Figure 1).

**Table 2:** Spearman's correlation analysis.

	**BMI**	**Leptin**	**Positive subscale**	**Negative subscale**	**General psychopathology subscale**	**Total**	**BPRS**
BMI	1.000						
Leptin	0.573^**^	1.000					
Positive subscale	−0.037	−0.113^*^	1.000				
Negative subscale	−0.159^**^	−0.187^**^	0.382^**^	1.000			
General psychopathology subscale	−0.085	−0.173^**^	0.712^**^	0.731^**^	1.000		
Total	−0.102	−0.192^**^	0.793^**^	0.798^**^	0.968^**^	1.000	
BPRS	−0.089	−0.177^**^	0.861^**^	0.677^**^	0.909^**^	0.949^**^	1.000

* *p-value < 0.05*.

** *p-value < 0.01*.

**Table 3:** Multiple stepwise regression analysis between leptin and psychopathology symptoms.

	**Coefficients**		***t***	***p-*value**	**95.0% confidence Interval for B**		**Collinearity statistics**		
	**B**	**Std. error**			**Lower bound**	**Upper bound**	**Tolerance**	**VIF**	**Minimum tolerance**
(Constant)	2.822	0.314	8.992	0.000	2.205	3.439			
Positive subscale	−0.071		−1.208	0.228			0.869	1.151	0.869
Negative subscale	−0.043	0.014	−3.102	0.002^*^	−0.070	−0.016	1.000	1.000	
General psychopathology subscale	−0.055		−0.704	0.482			0.486	2.056	0.486
PANSS total score	−0.093		−1.009	0.314			0.357	2.800	0.357
BPRS total score	−0.055		−0.745	0.457			0.545	1.835	0.545

* *Indicated the Bonferroni corrected p-value, p < 0.05*.

### Regression Analysis Between Leptin, BMI, and Negative Symptoms

Table 4 showed that BMI had a positive effect on serum leptin (B = 2.322, *t* = 9.557, *P* < 0.001) and a negative effect on PANSS-N scores (B = −0.303, *t* = −2.784, *P* = 0.006). After adding leptin to the model, the effect of BMI on PANSS-N scores disappeared (B = −0.186, *t* = −1.514, *P* = 0.131), indicating that leptin may be a predictor for the negative symptoms.

**Table 4:** Regression analysis between leptin, BMI, and negative symptoms.

	**Dependent**	**Independent(s)**	**B**	***t***	***p-*value**
b(YX)	(Y)	(X)	−0.303	−2.784	0.006
b(MX)	(M)	(X)	2.322	9.557	
b(YX,M)	(Y)	(X)	−0.186	−1.514	0.131
		(M)	−0.050	−2.031	0.043

*X, BMI; Y, Negative symptoms; M, Leptin*.

## Discussion

Weight gain caused by antipsychotics is related to psychopathological improvement. When the patient's weight increased by 7%, the PANSS score decreased by 12% (32). The BPRS score of patients with more than 7% weight gain decreased by 45.6%, while the BPRS score of the rest of the patients decreased by only 31.9% (33). Previous studies have found that the higher the BMI of Chinese patients with chronic schizophrenia, the higher the plasma orexin-A level and the less negative symptoms (34). Many studies have shown a significant relationship between antipsychotic-induced weight gain and good treatment outcomes (20, 35). For example, for clozapine and olanzapine, there is a significant correlation between weight gain and clinical response to these antipsychotics, but the reason is not clear (36, 37). Most studies have shown a correlation between antipsychotic-induced weight gain and treatment benefits based on the above definition. Lower baseline weight was associated with weight gain induced by antipsychotics (38). In studies of controlled baseline weight or BMI, 90% of studies showed a link between symptom improvement and weight gain (33).

Weight gain and improvement caused by antipsychotics may result from the pharmacodynamic properties of antipsychotics, but the relationship between them is not necessarily interdependent. It has also been reported that weight loss in patients with schizophrenia through life intervention and drug intervention does not affect the efficacy of antipsychotic drugs (39). In summary, there is a correlation between antipsychotic-induced weight gain and the therapeutic benefit (40). However, the direct cause-effect relationship between antipsychotic-induced weight gain and the therapeutic benefit is unclear, or weight gain is not an absolute requirement for clinical efficacy.

In this study, serum leptin was negatively correlated with positive, negative, general pathological symptoms and PANSS total BPRS score in patients with schizophrenia. Multiple stepwise regression analyses showed that serum leptin level was significantly correlated with negative symptoms. In summary, this study showed that serum leptin levels in patients with schizophrenia were negatively correlated with the three subscales of PANSS (PANSS-P, PANSS-N, PANSS-G), the total scale score, and the BPRS score. This result is consistent with the reported changes in serum leptin levels and the severity of negative symptoms (41, 42). A large body of evidence suggests that dopamine system dysfunction is associated with negative symptoms of schizophrenia, leading to a lack of will and pleasure (43–45). More studies have shown that the role of leptin in protecting cell survival, promoting apoptosis, and dopamine regulation may be the main mechanism for improving the psychopathological symptoms of schizophrenia (46). A previous study found that plasma leptin levels were negatively correlated with PANSS depression factor scores (*r* = −0.255, Bonferroni corrected *P* = 0.028) (47). These findings support the hypothesis that leptin is a predictor of the reduction of negative symptoms in schizophrenia.

If leptin can relieve psychopathological symptoms, then weight gain in patients with schizophrenia is of concern. The leptin level in cerebrospinal fluid has been positively correlated with plasma leptin level and BMI (48). Another study found that leptin levels in schizophrenic patients treated with antipsychotics were higher than those in healthy controls, which was associated with weight gain caused by antipsychotics (49, 50). In addition, studies on patients without medication have shown that low leptin levels are associated with schizophrenia, and antipsychotics will increase leptin levels. Therefore, it is beneficial to study the relationship between leptin, BMI, and psychopathological symptoms. Interestingly, there were no statistically significant differences in age of onset, disease duration, type or the dose of antipsychotics between the high-leptin and low-leptin groups. It suggests that the antipsychotic was not the focus of the study.

The serum leptin level in women (3.24 ± 2.07 ng/ml) was higher than that in men (0.97 ± 1.05 ng/ml). There are three possible reasons for this phenomenon: Firstly, the location of fat deposition and the proportion of fat in the body (51). Subcutaneous fat is dominant in females, while visceral fat is more abundant in males. Subcutaneous fat produces more leptin than visceral fat; therefore, males' serum leptin levels are lower than females' (52). Another possibility is the effect of sex hormones: oestradiol promotes the secretion and release of leptin in cultured adipose tissue, but this process does not occur in men (53). Testosterone has been shown to have antiregulatory effects, suggesting that males' leptin concentrations may be lower than those in females, even when body fat ratios are similar (54, 55). Finally, it is possible that leptin production is higher in the female brain than in the male brain, leading to higher circulating leptin levels in female blood (56, 57).

Several limitations of this study need to be emphasized. Firstly, due to the cross-sectional design, this study cannot obtain causality. These findings must be carefully interpreted and explained in future longitudinal studies. Secondly, we focused on the effect of leptin on weight gain and included other relevant indicators, such as lipoprotein and triglyceride. In addition, leptin gene and leptin receptor gene polymorphisms may affect antipsychotic drug-induced weight gain. However, our study did not evaluate the effect of leptin receptor gene polymorphism on prognosis. Finally, we believe that different kinds of antipsychotics may have different effects on body weight. Therefore, further research is needed to evaluate and control these effects.

## Conclusion

It is the first study to suggest that leptin may play a role in the relationship between antipsychotics-induced weight gain and favorable treatment response in schizophrenia. These findings help us further understand the relationship between leptin, weight gain, and antipsychotic response in patients with schizophrenia. Our findings may help monitor the recovery of patients with schizophrenia and develop new treatment options.

## Data Availability Statement

The datasets presented in this study can be found in online repositories. The names of the repository/repositories and accession number(s) can be found in the article/supplementary material.

## Ethics Statement

The studies involving human participants were reviewed and approved by the Ethics Committee of Chaohu Hospital affiliated with Anhui Medical University. The patients/participants provided their written informed consent to participate in this study.

## Author Contributions

YeZ, YY, and XN collected and analyzed the data. KZ and HL gave administrative support. YeZ and XL wrote the paper. YuZ, XY, YY, TZ, and LX provided insightful discussion for the manuscript. All authors contributed to data interpretation and approved the final version for publication.

## Funding

This research was supported by grants from the National Natural Science Foundation of China (Grant No. 81771449) and the Anhui Province Key Scientific and Technological Projects (1804h08020263).

## Conflict of Interest

The authors declare that the research was conducted in the absence of any commercial or financial relationships that could be construed as a potential conflict of interest.

## Publisher's Note

All claims expressed in this article are solely those of the authors and do not necessarily represent those of their affiliated organizations, or those of the publisher, the editors and the reviewers. Any product that may be evaluated in this article, or claim that may be made by its manufacturer, is not guaranteed or endorsed by the publisher.
